# Supplementary material for: The pattern of genetic variability in a core collection of 2,021 cowpea accessions
Source: G3 (Bethesda). 2024 May 6;14(6):jkae071. doi: 10.1093/g3journal/jkae071 (PMC11152079; doi:10.1093/g3journal/jkae071)
Supplement: jkae071_Supplementary_Data [file jkae071_supplementary_data.zip › Supplemental_Figures_G3-2024-404821.pdf]

Supplemental Figures for

The pattern of genetic variability in a core collection of 2,021 cowpea accessions

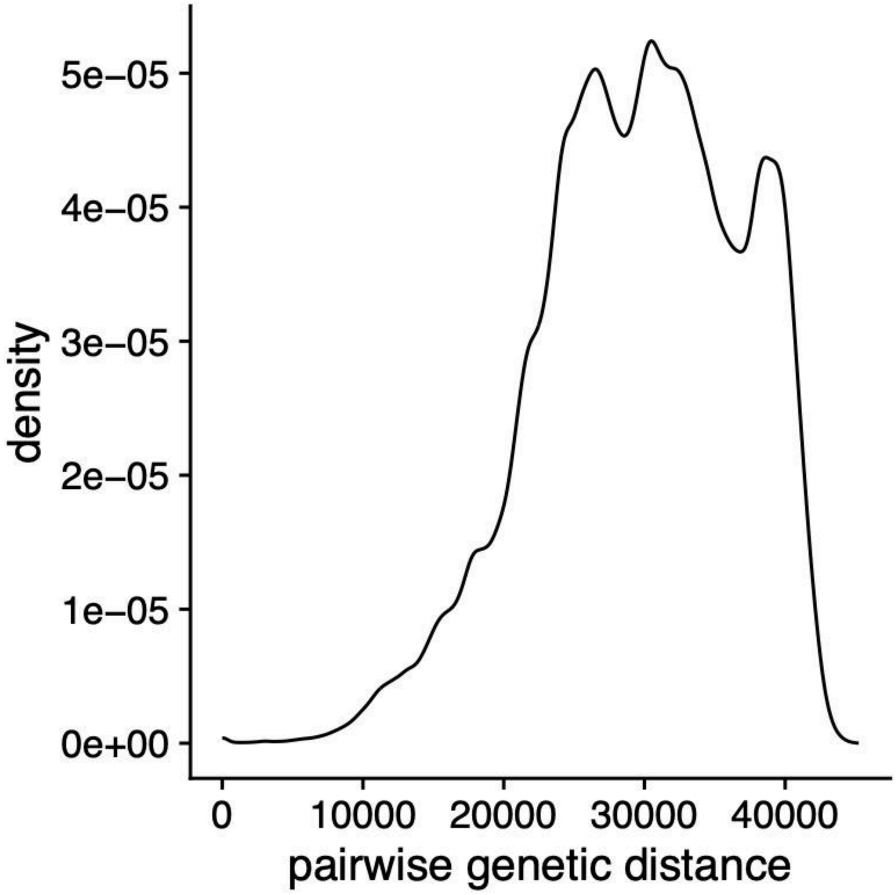

Figure S1. Distribution of pairwise genetic distances between accessions.

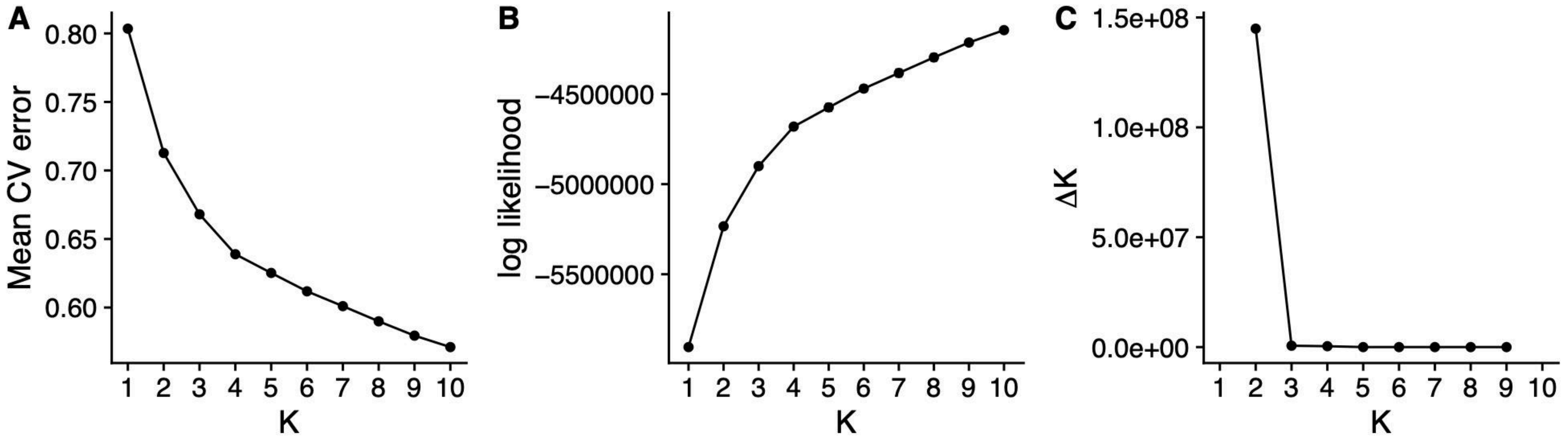

Figure S2. Admixture statistics per K.
A) Mean cross-validation error. B) Mean log-likelihood. C) Delta K.

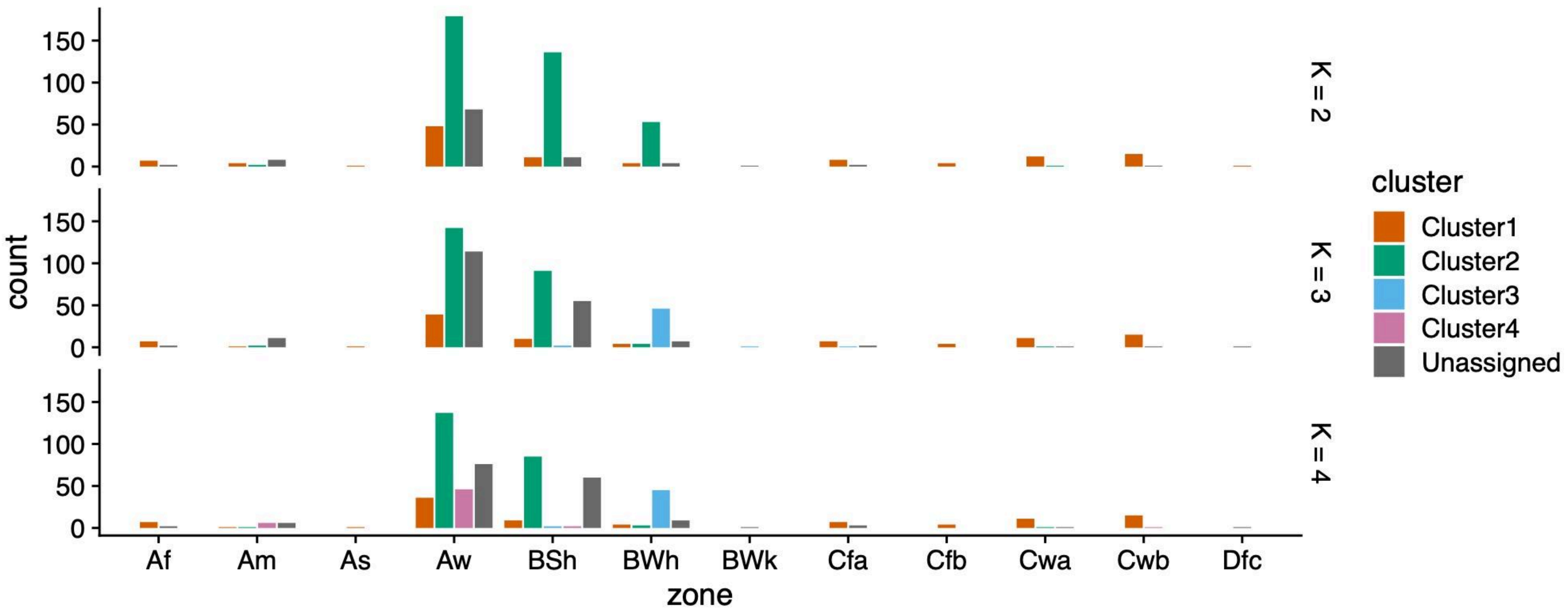

Figure S3. Climate zone distribution by subpopulation cluster for accessions with collection coordinates. Climate designations correspond to the Köppen climate classification.

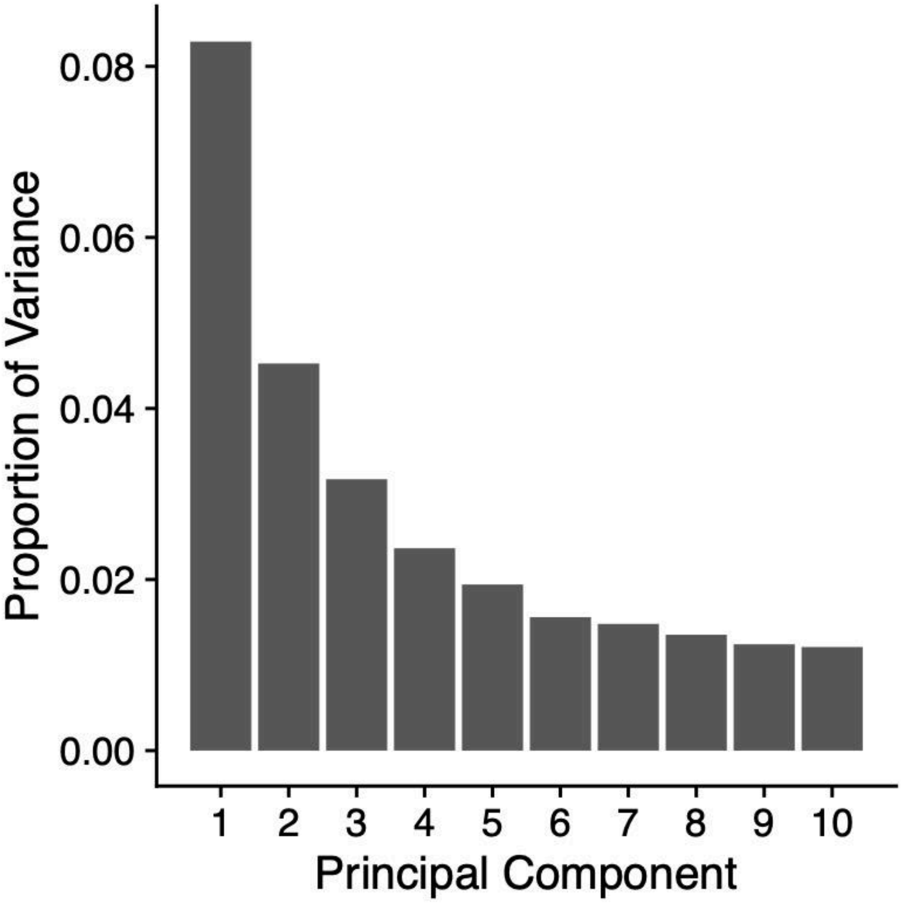

Figure S4. Proportion of variance explained by the first ten principal components.

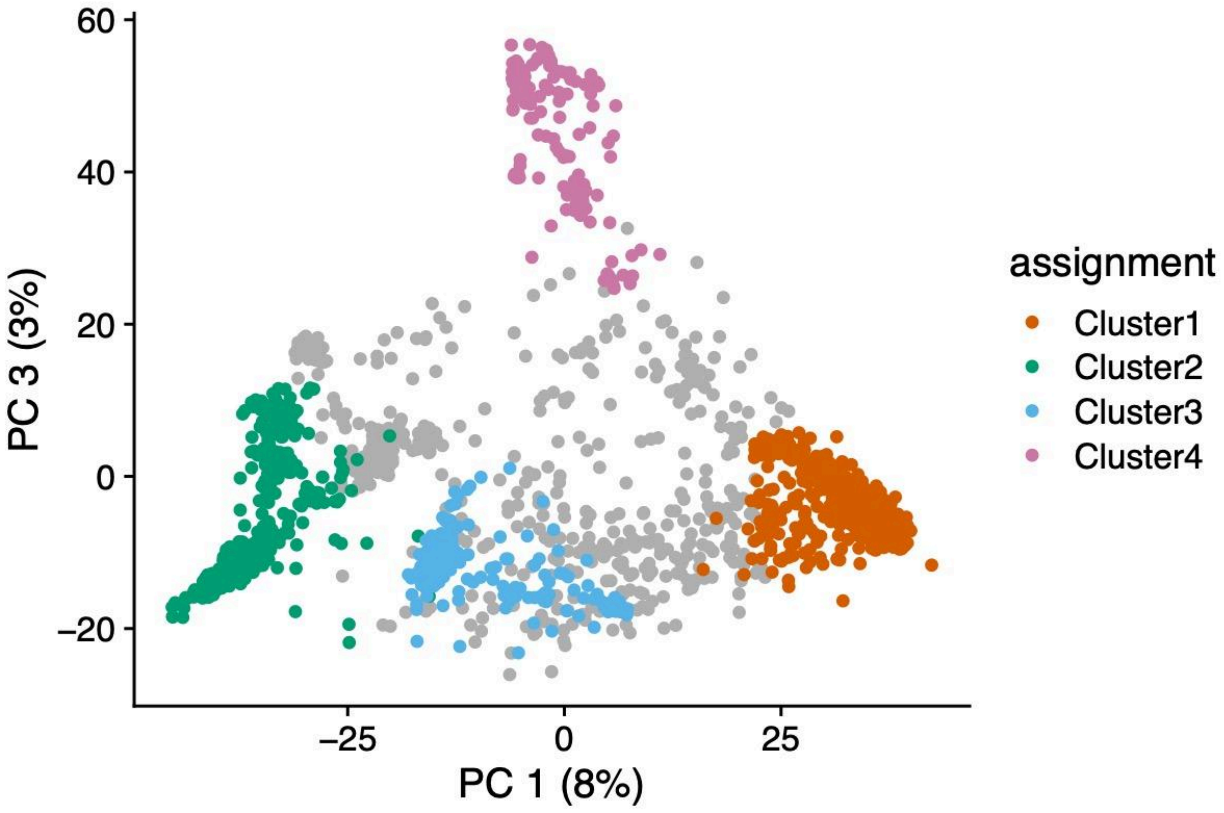

Figure S5. PC 1 vs. PC 3 (K = 4).

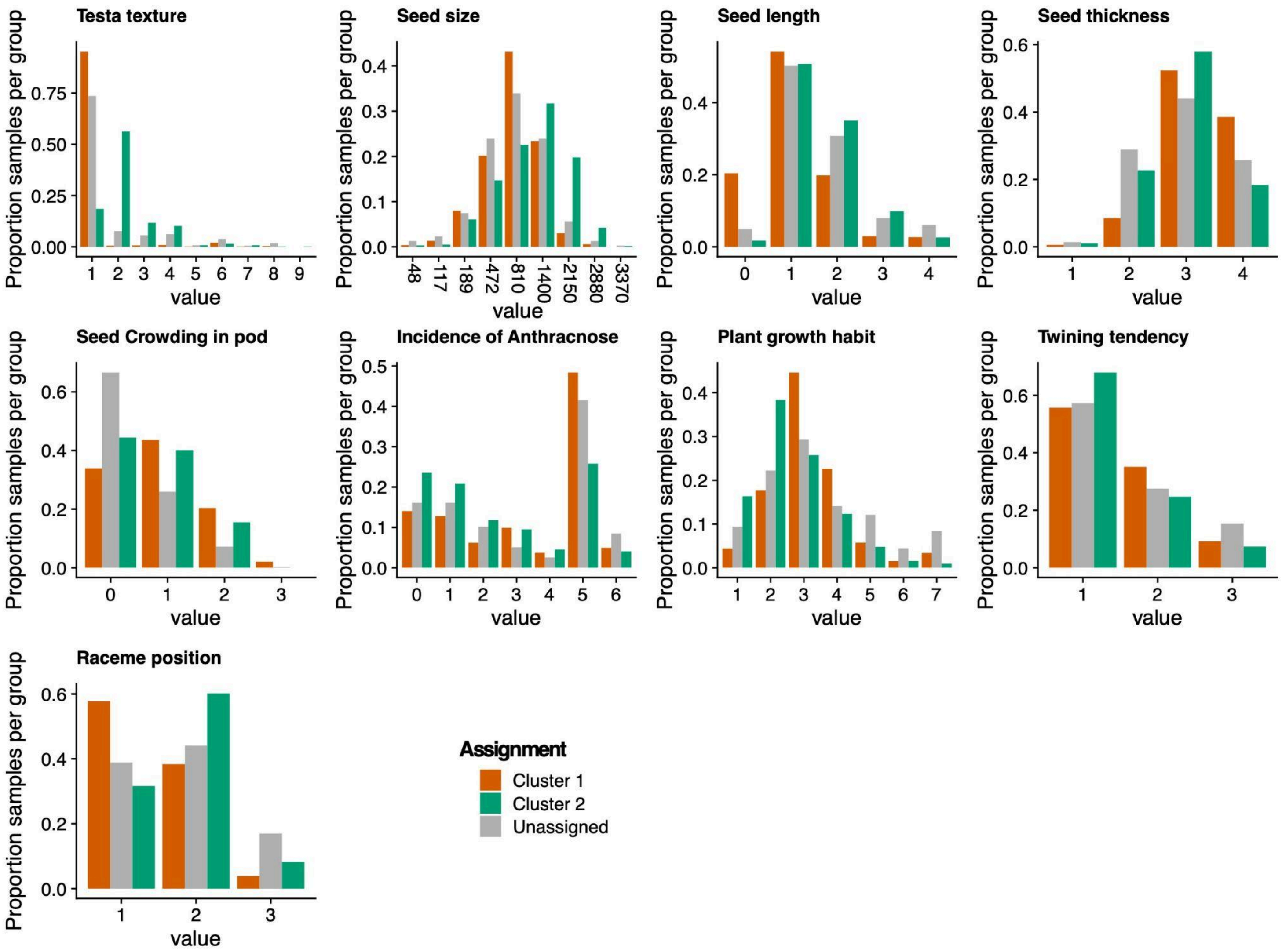

Figure S6. Distributions of discrete phenotypes that vary between Cluster 1 and Cluster 2 (K = 2). All phenotypes shown had a statistically significant difference between the two subpopulation clusters (Mann Whitney Wilcoxon adjusted p-value < 0.05).

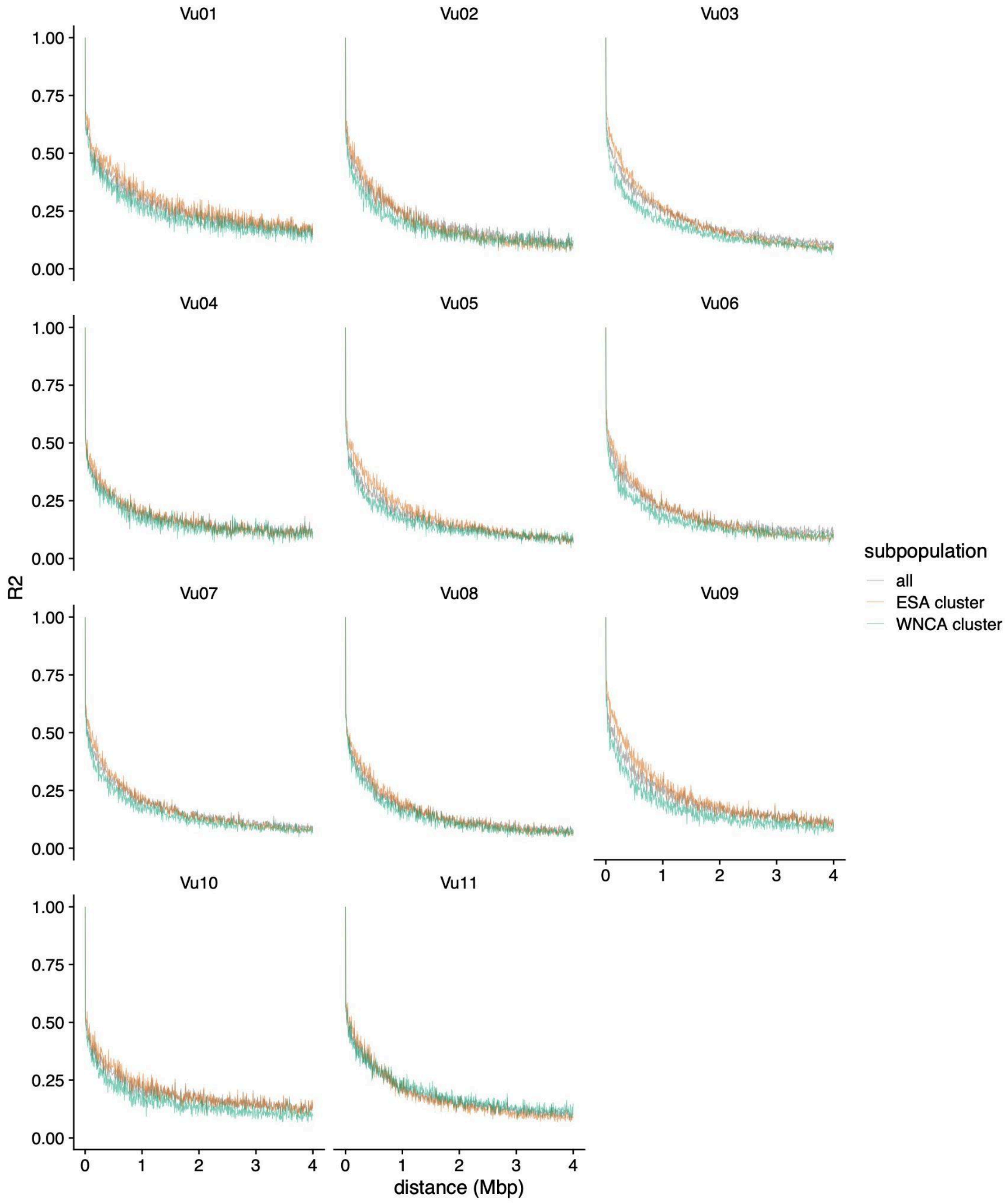

Figure S7. Linkage disequilibrium decay by chromosome.

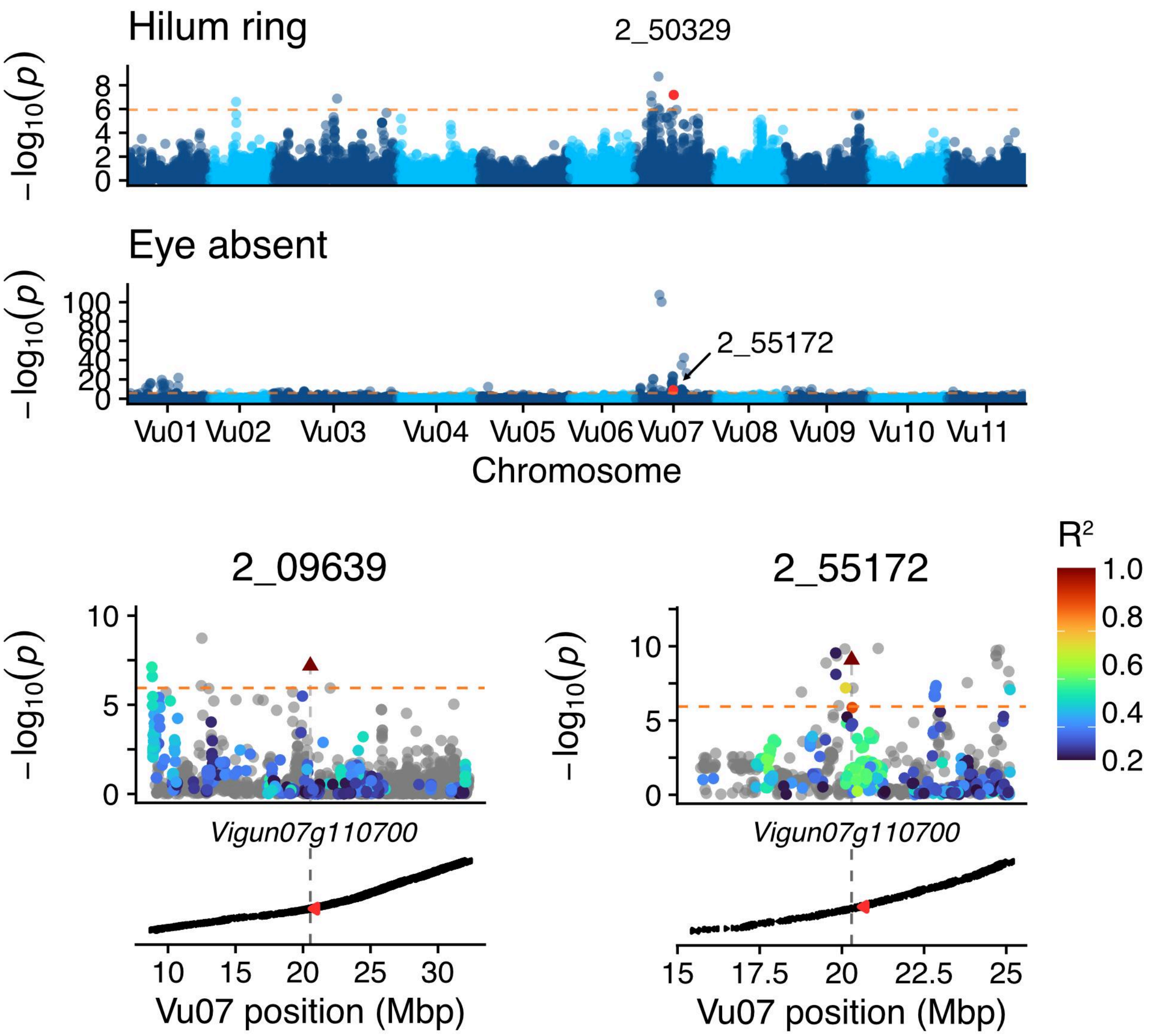

Figure S8. Genome-wide association mapping manhattan plots and focal loci for additional eye pattern phenotypes.

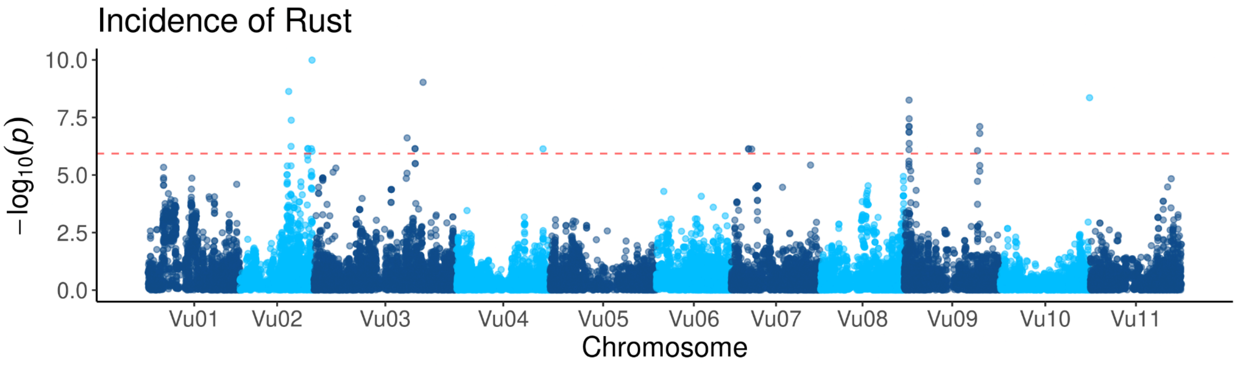

Figure S9. GWAS for rust resistance

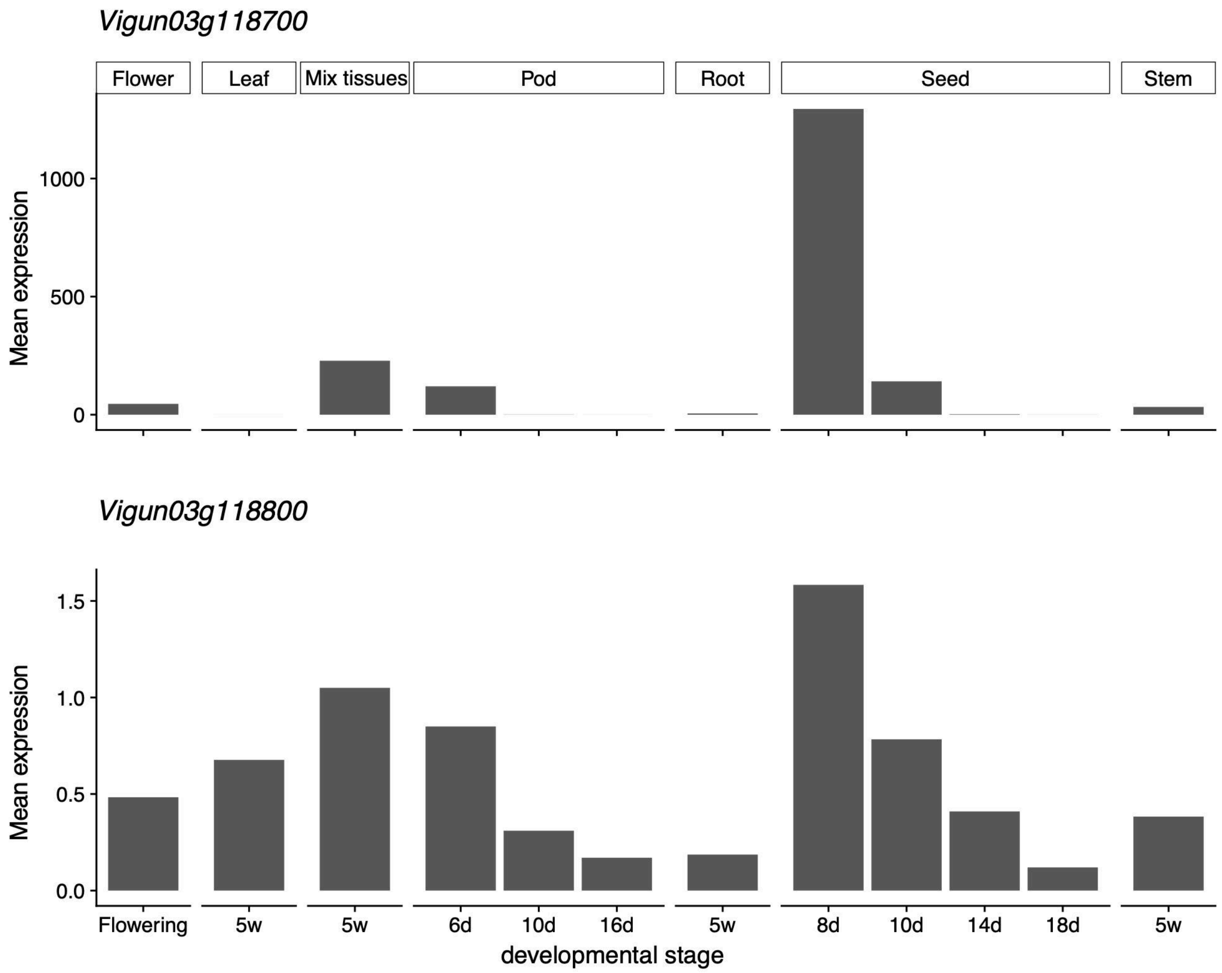

Figure S10. Expression of 2 candidate genes for Tan and Red seed coat color across developmental stages and tissues.
